# Experimental subaqueous burial of a bird carcass and compaction of plumage

**DOI:** 10.1007/s12542-018-0411-y

**Published:** 2018-06-11

**Authors:** Evan T. Saitta, Charles Clapham, Jakob Vinther

**Affiliations:** 1 School of Earth Sciences, University of Bristol, Wills Memorial Building, Queens Rd, Bristol, BS8 1RJ UK; 2 School of Biological Sciences, University of Bristol, Life Sciences Building, Tyndall Avenue, Bristol, BS8 1TQ UK; 3 Integrative Research Center, Section of Earth Sciences, Field Museum of Natural History, 1400 S. Lake Shore Dr., Chicago, IL 60605 USA

**Keywords:** Taphonomy, Feather, Fossil, Burial

## Abstract

‘Exceptional fossils’ of dinosaurs preserving feathers have radically changed the way we view their paleobiology and the evolution of birds. Understanding how such soft tissues preserve is imperative to accurately interpreting the morphology of fossil feathers. Experimental taphonomy has been integral to such investigations. One such experiment used a printing press to mimic compaction, done subaerially and without sediment burial, and concluded that the leaking of bodily fluid could lead to the clumping of feathers by causing barbs to stick together such that they superficially resemble simpler, less derived, filamentous structures. Here we use a novel, custom-built experimental setup to more accurately mimic subaqueous burial and compaction under low-energy, fine-grain depositional environments applicable to the taphonomic settings most plumage-preserving ‘exceptional fossils’ are found in. We find that when submerged and subsequently buried and compacted, feathers do not clump together and they maintain their original arrangement. Submersion in fluid in and of itself does not lead to clumping of barbs; this would only occur upon pulling feathers out from water into air. Furthermore, sediment encases the feathers, fixing them in place during compaction. Thus, feather clumping that leads to erroneously plesiomorphic morphological interpretations may not be a taphonomic factor of concern when examining fossil feathers. Our current methodology is amenable to further improvements that will continue to more accurately mimic subaqueous burial and compaction, allowing for various hypothesis testing.

**Electronic supplementary material:**

The online version of this article (10.1007/s12542-018-0411-y) contains supplementary material, which is available to authorized users.

## Introduction

‘Exceptional fossils’ of feathered dinosaurs, particularly those discovered over the last two and a half decades, have radically changed our views of bird and feather evolution (Ostrom 1976; Norell and Xu 2005; Zelenitsky et al. 2012; Godefroit et al. 2014; Xing et al. 2016) and have provided the molecular fossils, namely melanosomes, needed to reconstruct the coloration of dinosaurs (Vinther 2015), providing insight into their paleobiology. Given the scientific implications such data from these soft tissue-bearing fossils hold, recent interest has emerged regarding the mechanisms of their preservation. While some studies chemically analyze the fossils themselves (Field et al. 2013), others use experimental taphonomy to understand what components of keratinous structures persist into the fossil record (Saitta et al. 2017).

One such example of using experimentation to study the taphonomy of feathers involved crushing bird carcasses in a printing press subaerially and without sediment burial (Foth 2012). The bodily fluids that leaked out caused feather barbs to clump together, superficially resembling simpler, filamentous structures that lack the high-order branching seen in many modern feathers. Such taphonomic clumping as a result of compaction might lead relatively morphologically complex feathers to be misinterpreted as simpler, more basal structures. This would be an important taphonomic bias, as the common model of feather evolution involves increasingly higher order branching structures deriving from simpler filaments (Prum 1999; Prum and Brush 2002). Accurate morphological descriptions of fossil feathers are important given that some morphotypes appear to differ from those observed in modern feathers or predicted from an evo–devo model (Zhang et al. 2008; Xu et al. 2009; O’Connor et al. 2012). Some of these morphotypes, such as those that appear to show thick, fused, ribbon-like regions, were suggested to be a taphonomic artefacts of this feather barb clumping (Foth 2012). However, we doubt whether inducing the escape of bodily fluid using a printing press is an accurate simulation of the burial and compaction experienced by exceptional fossils preserving plumage, which are often found in low-energy, fine-grain, aquatic depositional settings (Norell and Xu 2005). We hypothesize that submersion in water and encasement in sediment should preserve barb orientation and limit clumping as a result of escaping bodily fluids during compaction. Here, we attempt to more accurately simulate subaqueous burial and compaction using custom-built experimental equipment in order to test if such previously described feather clumping occurs when fully submerged and subsequently encased in sediment prior to compaction. One carcass was subaqueously buried and compacted before exhumation and analysis, while a second carcass underwent a decay treatment after subaqueous burial and prior to compaction, exhumation, and analysis. We do not attempt to precisely mimic all of the physicochemical burial conditions of any particular fossil locality, but rather attempt to examine subaqueous burial and compaction more generally.

## Methods

Two small zebra finches (*Taeniopygia guttata*) were purchased alive. Work with animals was approved by the UK Home Office and both were euthanized according to Schedule One of the Animals (Scientific Procedures) Act of 1986 through asphyxiation. Carcasses were stored in a freezer until experimental treatments were performed.

**Fig. 1:**
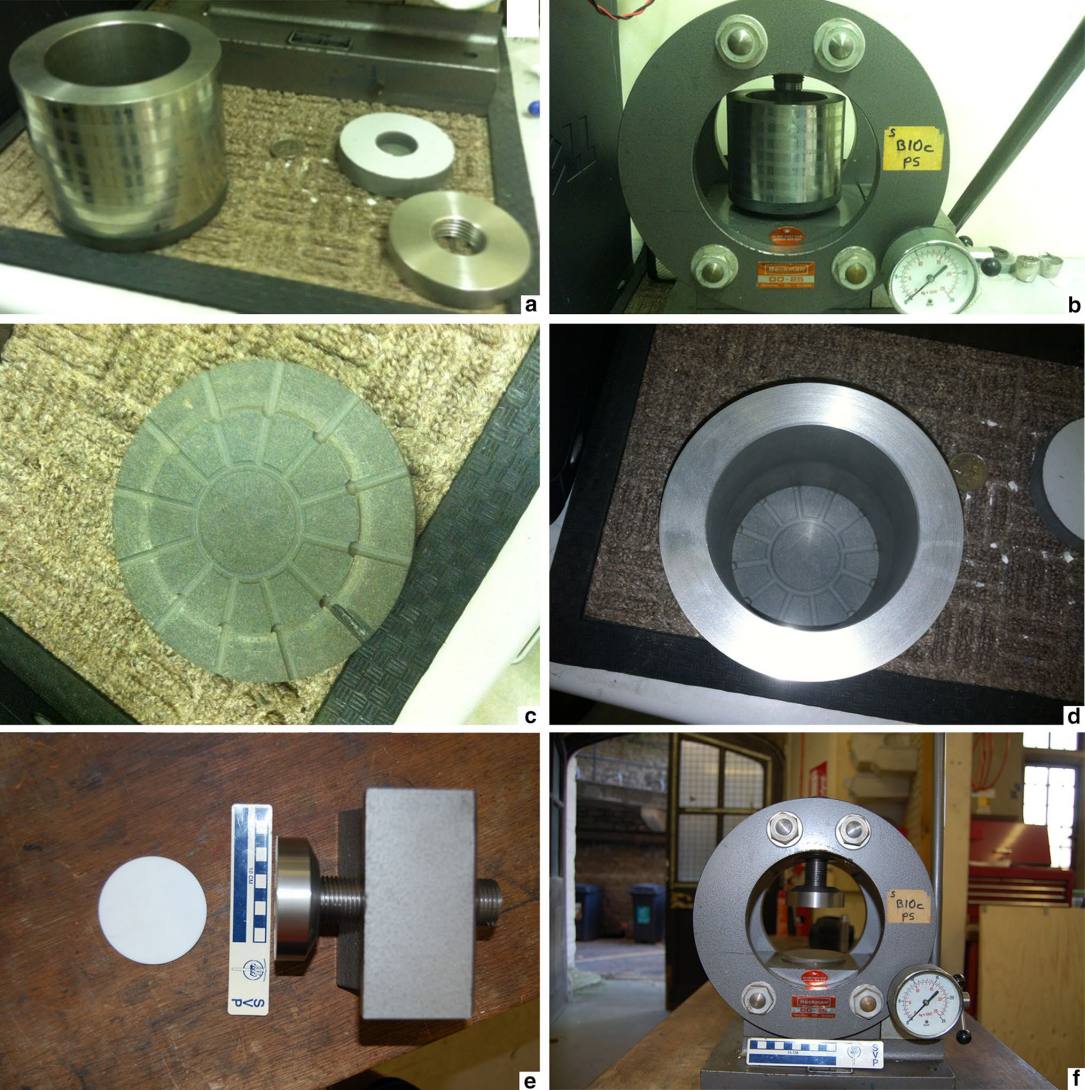
Compaction rig. **a**–**d** First version. **e**–**f** Second version. **a** Chamber, thick Trespa disk, and stainless steel spacing ring (*from left to right*). **b** Chamber placed in hydraulic press (but without disk and ring threaded onto the frame). **c** Trespa base with grooves. **d** Chamber sitting on grooved, Trespa base. **e** Thin PTFE disk and permanently attached stainless steel plunger threaded onto a subcomponent of main frame. **f** Subcomponent with permanently attached plunger bolted to the press

A subaqueous compaction rig (Fig. 1) consisted of a 9 cm tall chamber that can be placed into a hydraulic press. The chamber was a stainless steel cylinder with an 8 cm internal diameter. It sat on a specially designed base made of Trespa with radial grooves allowing water to escape from the bottom/sides during compaction (8 cm diameter, 13.5 mm maximum thickness). To load the chamber, sewing thread was passed into the chamber through two opposite grooves on the base. Several centimeters of sand/chalk/soil mixture were then added to the bottom. Bowland Stone Essentials Range sharp sand with grain size of typically about 0.5 mm (although variation results in a mixture of mostly medium sand grains and some coarse sand grains) was obtained from a garden shop in Bristol, UK. Chalk powder was purchased online. Soil containing organics was obtained from the Clifton Downs park in Bristol, UK. All mixtures comprised roughly equal parts.

For the first experimental setup, a finch was slid under the thread, and slack in the thread was removed by pulling on the ends outside the chamber in order to fasten the bird to the sediment and prevent floating. The junction between the chamber and the base was sealed with adhesive tape, keeping the thread taut. Tap water was then added. Next, a sieve was used to add a chalk/soil mixture to the water in order to replicate low-energy, gradual burial.

After the chamber was loaded, the tape was removed, and water was allowed to flow out into a plastic bag under gravity. A thick Trespa disk was fitted snugly into the top of the cylindrical chamber with a central concavity to accommodate the main frame of the hydraulic press (~ 8 cm diameter, 16 mm maximum thickness). To add stability, a stainless steel spacing ring (the same diameter as and a similar thickness to the thick Trespa disk) with internal threading (so as to allow it to be threaded onto the main frame of the press) was placed directly on top of the thick Trespa disk. The chamber, still in the plastic bag, was placed into the press, and the spacing ring was threaded onto the main frame of the press by spinning the chamber. The base of the press, on which the chamber sat, was raised using a manual crank, compacting the sediment column and bird within the chamber. Compaction was monitored using a built-in pressure gauge. Compaction was increased to approximately 25 tonnes, translating to 48,769,892.6 Pa of pressure (held for 1 min), in an attempt to sufficiently compact the sediment into a consolidated block. Water escaping from pore spaces during compaction flowed out freely from the specially designed, grooved, Trespa base, while almost all sediment was retained.

The chamber was taken out of the press and the sediment column was plunged out of the cylinder. The sediment column was placed into a fume hood for 6 days and then placed into an oven for 24 h at 60 °C to more rapidly dry the sediment. The column was then prepped using compressed air (without abrasive) and a dental pick. Photos were taken throughout the prepping process with a Nikon D90 DSLR camera (see supplementary material).

The rig was set up identically for the second experiment with the following modifications. First, above the basal sand/chalk/soil mixture, chalk/soil mixture was added prior to adding the carcass and water so that the carcass would be completely surrounded by a muddy mixture. Soil was intended to introduce decay microbes. Second, the upper opening of the loaded chamber (with water added, bird buried, and bottom taped) was partially sealed with parafilm. The whole chamber was then placed into a plastic bag that was tied off at the top. The chamber was placed into an incubator at ~ 37 °C for 46 days (although a power outage of unknown duration caused temperatures to drop for several days during this time). A day after the incubation period ended, a dry chalk/soil/sand mixture (of roughly equal parts) was added to rebury the detached head and part of the rectrices that had floated to the top of the column at some point during incubation, followed by pure sand as a cap (to prevent excessive suspension of sediment upon subsequent hydration). The whole chamber was then submerged in tap water and the sediment was probed in order to facilitate rehydration of the sediment, much of which had dried out during incubation. After a day of submergence, the chamber was removed and compacted. Third, a stainless steel plunger (26-mm-thick head at the end of a threaded rod) was permanently attached to the main frame of the press and the thick Trespa disk was replaced with a thinner polytetrafluoroethylene (PTFE) disk (~ 8 cm diameter, 3 mm thickness). Fourth, after compaction, the sediment column was left in a fume hood for 3 days before placing it in the oven to accelerate drying.

## Results

The compaction rig effectively consolidated the sediment in both runs into a stable column, roughly the consistency of a block of chalk, capable of being manipulated by hand.

**Fig. 2:**
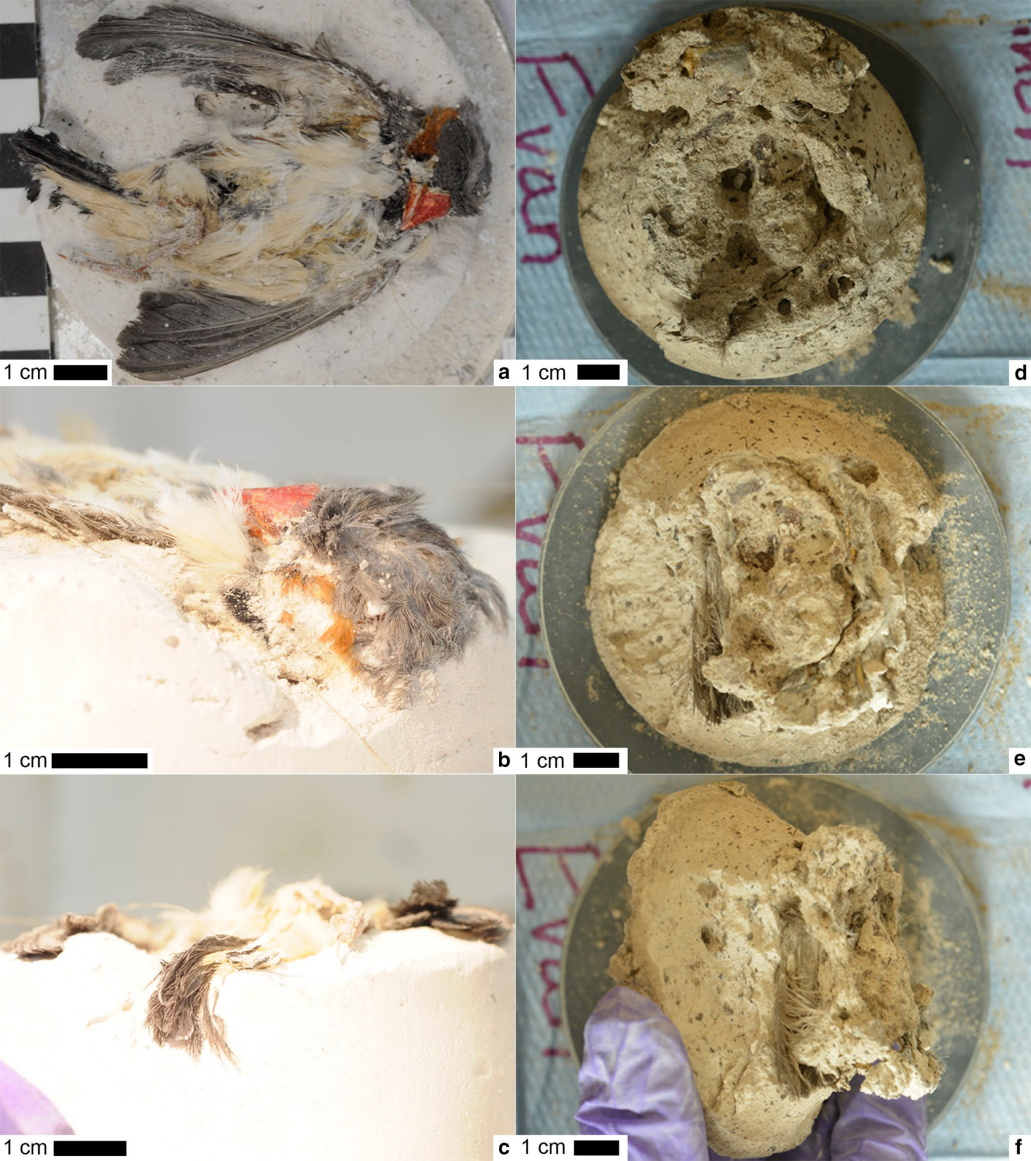
Preparation of the sediment columns after compaction. **a**–**c** The non decayed bird after compaction. **a** Ventral view. **b** Lateral view of head. **c** Posterior view of rectrices. **d**–**f** The decayed bird after compaction. **d** Overhead view of column early in preparation (high stratigraphically) with the head still present. **e** Overhead view of the column later in preparation (low stratigraphically) with the head removed. **f** Lower stratigraphic layer in side view, showing remiges

The compacted non decayed finch (Fig. 2a–c) exhibited some crushing of the torso but retained much of its three-dimensionality, especially in the head. Escaped body fluids resulted in some feathers adhering to the sediment. No signs of feather clumping across the body or among different feather types were seen, with the original feather morphology, orientation, and configuration still observable.

In the compacted decayed finch (Fig. 2d–f), the skull and rectrices separated from the body and floated up through the sediment prior to the sediment drying out, showing the best preservation of any of the regions of the carcass. The head remained three-dimensional, with the orange beak coloration preserved. The rest of the carcass that did not float up was highly degraded. Many small, unidentifiable remnants of the carcass were dispersed through the sediment column. The only anatomical features identifiable at a low stratigraphic level in the column were portions of remiges that were slightly browned in color. Not even bones were apparent.

## Discussion

The results from the compacted non decayed finch show that compaction after subaqueous burial does not greatly alter the appearance of the feather morphology. Therefore, the results of Foth (2012) appear to derive from unrealistic simulation of the taphonomic conditions (i.e., subaerial and without sediment burial). The use of a printing press allows bodily fluids to escape from the carcass, and when this occurs subaerially, such fluids bind feather barbs together and may lead to the appearance of a more primitive morphology. The more realistic taphonomic simulation reported here shows that feather morphology is likely preserved through burial and compaction, and that complex feathers do not clump to look like proto-feathers. Many exceptional fossil feathers and plumage-preserving taxa are found in low-energy aquatic depositional environments (Norell and Xu 2005), so our experimental results provide useful comparisons even if they are not precise replications of the exact physicochemical burial environment of such fossils. Burial is accelerated experimentally here by increasing the rate of sedimentation while keeping the energy of the grains low using a sieve. Plumage fully submerged in water does not clump together, as is the case when feathers are dry. Rather, the introduction of fluids subaerially clumps barbs together. Furthermore, encasing sediment fixes the plumage into place and secures the position of such tissues through early compaction. This matches what we see in fossils, where lateral expansion is not observed due to decay or compaction, with the exception of certain rigid mineralized tissues. Therefore, compression fossils, such as those with carbonaceously preserved soft tissues, provide two-dimensional views of what were once three-dimensional organisms (Briggs and Williams 1981). Any preserved manipulation of feather barbs would have to occur prior to burial, since the encasing sediment can secure tissues into place, and simply being submerged in water does not cause barbules to clump together. With the limited pressures used in our experiments, our results do not produce fully flattened carcasses as seen in such compression fossils, and are more appropriate to early burial conditions, especially since diagenetic heat, which would lead to volume loss from labile tissues, is not concurrently simulated here.

The compacted decayed finch provided limited data since the decay process had proceeded further than expected and little of the carcass remained. However, the survival of some remiges suggests that these feathers might be able to resist microbial/autolytic decay longer than other feathers and tissues do in certain environmental conditions. Such survival may possibly be due to factors such as calcification (Pautard 1963, 1964), melanization (Gunderson et al. 2008), surface lipid composition, or the larger mass of keratin protein than in smaller feathers or other tissues, keratin being fairly recalcitrant compared to other proteins (Fraser et al. 1972). Perhaps this sample experienced similar burial conditions to those of ‘bog bodies’ (i.e., an acidic and reducing environment), which also show keratin preservation at the expense of bone preservation (Evershed 1992).

Our methodology represents an improvement from Foth (2012) in studying early subaqueous burial and compaction in an experimental framework. However, further changes may continue to improve upon this physical modeling of early taphonomic processes to provide more realistic results applicable to fossils. For one, future experiments can allow the bird carcass to ‘bloat and float’ until the body cavity ruptures and the carcass sinks to the sediment, rather than accelerating the process by securing the carcass to the sediment–water interface. Increasing compaction forces will also lead to more flattening than the minimal crushing observed here. Finally, subaqueous burial and compaction should be linked to thermal maturation in order to induce chemical degradation comparable to diagenesis.

## Conclusion

Early attempts to mimic the taphonomic compaction of plumage used unrealistic simulations (i.e., subaerial and without sediment burial), leading to feathers clumping to resemble simpler, more plesiomorphic morphologies. Here, more realistic simulation of subaqueous burial and compaction using custom-built experimental equipment shows that the appearance of feather morphology is not altered during such taphonomic processes; submerged barbs are not clumped together and sediment encases the soft tissues, fixing them in place. Given that many ‘exceptional fossils’ showing plumage preservation underwent such low-energy subaqueous burials, our results are applicable to the fossil record. Thus, interpretations of fossil feather morphologies do not require too much concern regarding taphonomic clumping of feathers. Fossil feathers interpreted as simple, plesiomorphic morphotypes are likely genuine rather than taphonomically altered. Further advancements in the methodology promise ever more realistic simulations of subaqueous burial and compaction, and additional experiments will allow for greater disentanglement of the many variables involved in such taphonomic settings.

## Electronic supplementary material

Below is the link to the electronic supplementary material.

Supplementary material 1 (M4V 4518 kb)
